# Comparison of hyaluronic acid, chlorhexidine and persica during SRP

**DOI:** 10.6026/973206300221212

**Published:** 2026-02-28

**Authors:** Neeraj Chandra, Mutyala Swati, Lawanya Chandrakar, Akshay A Katara, Astha Shrivastava, Dharmen Bhansali

**Affiliations:** 1 Department of Periodontics, Institute of Dental sciences, Bareilly, Uttar Pradesh, India; 2 Department of Dental Surgery, Surabhi Institute of Medical Sciences, Mundrai, Telangana, India; 3 Department of Periodontics, Shri Balaji Institute of dental science, Raipur, Chhattisgarh, India; 4 Department of Dental Surgery, Specialist Dentist (Orofacial Pain), Dentistry, Omni Family Health, Bakersfield, California, United States; 5 Department of Oral Medicine, Diagnosis and Radiology, Rungta College of Dental Sciences and Research, Bhilai, Chhattisgarh, India

**Keywords:** Hyaluronic acid (HA), chlorhexidine (CHX), *Salvadora persica* (SP), scaling and root planing (SRP), gingivitis, anti-inflammatory

## Abstract

The optimal choice of local drug delivery agents for chronic periodontitis treatment remains uncertain. This study compared 0.8% hyaluronic acid (HA), 0.2% chlorhexidine (CHX) and 10% *Salvadora persica* (SP) gels versus SRP alone in stage I and II chronic periodontitis patients. 100 patients were randomized into four groups: HA gel, CHX gel, SP gel and SRP alone. Results showed significant clinical improvement with HA showing superior wound healing, CHX having the best antimicrobial effects but higher side effects and SP demonstrating minimal side effects with adequate antimicrobial potential. This study contributes to the understanding of the efficacy and safety of different local drug delivery agents, offering guidance for periodontists in clinical practice.

## Background:

Chronic periodontitis affects 10-15% globally, causing progressive periodontal loss due to inflammation caused by biofilm presence [1]. Scaling and root planing (SRP) remains the gold-standard treatment; however, residual biofilms and refractory inflammation persist in some patients [3]. Adjunctive topical agents- chlorhexidine (CHX, antimicrobial-predominant), hyaluronic acid (HA, regenerative-predominant) and *Salvadora persica* (SP, balanced antimicrobial/anti-inflammatory) have been evaluated as SRP adjuncts [2 ]. Chlorhexidine has been a gold standard in the form of adjunctive treatment however, the local delivery do have its own advantages as well as disadvantages. It still is considered to the positive control for any study in order to reduce bias. HA has been gaining favors recently due to its anti-microbial as well improved wound healing and bone healing properties [3]. At the same time the inclination towards herbal products have shown that SP to be effective microcidal and inflammation modulating agent. Previous comparative studies have demonstrated potential methodological bias by comparing agents with disparate mechanisms without including balanced comparators [4]. Recent meta-analytical evidence confirms superior efficacy of HA when combined with SRP in chronic periodontitis management [5] thus, the present study was designed in order to incorporate positive and negative control to assess one anti-inflammatory and on anti-microbial component as both have significant effect on periodontitis mechanism. Therefore, it is of interest to evaluate the efficacy and safety of adjunctive topical agents, including chlorhexidine, hyaluronic acid and *Salvadora persica*, in chronic periodontitis treatment, providing balanced comparisons between antimicrobial and regenerative agents.

## Methodology:

This prospective, randomized, double-blind, four-arm RCT enrolled 100 systemically healthy patients (age 25-60 years) with generalized plaque-induced gingivitis or stage I-II periodontitis (n = 25/group: HA, CHX, SP, control). Inclusion criteria: ≥20 natural teeth, full-mouth BOP ≥25%, no periodontal treatment within 6 months. Exclusion criteria: systemic disease, smoking >10 pack-years, allergy to study components. IRB approval was obtained; informed consent was provided by all participants. Computer-generated block randomization was done to assign participants to groups. Patients, clinical examiners and microbiological analysts were blinded. The treating periodontists remained unblinded for gel application only. All groups received standardized SRP using ultrasonic instruments and hand instruments completed in 2 sessions. All patients received oral hygiene instruction.

[1] Group 1: 0.8% hyaluronic acid gel (Gengigel®) applied 3 times daily x 7 days, then once daily x 14 days

[2] Group 2: 0.2% chlorhexidine gel (Perio-Protect®) using identical protocol

[3] Group 3: 10% standardized *Salvadora persica* gel extract (alkaloid content ≥0.8%, verified via HPLC-UV) using identical protocol

[4] Control Group: SRP alone without adjunctive gel

Clinical parameters were measured at baseline and 2 months by a calibrated, blinded examiner using UNC-15 periodontal probe (0.5 mm accuracy): Plaque Index (PI - Modified Löe & Silness): (0-3 scale), Gingival Index (GI - Löe & Silness), Bleeding on Probing (BOP): Dichotomous; expressed as percentage, Probing Pocket Depth (PPD): Six sites per tooth (mm), Clinical Attachment Level (CAL): Six sites per tooth (mm), Intra-examiner reproducibility (ICC) was 0.91-0.97 for all parameters. Microbiological Analysis: Subgingival samples were collected at baseline and 72 hours post-treatment from the deepest pocket using sterile ISO #40 paper points. Samples were cultured under anaerobic conditions (85% N_2_, 10% CO_2_, 5% H_2_) at 35°C for 7-14 days on selective media. *Porphyromonas gingivalis*, *Fusobacterium nucleatum*, *Tannerella forsythia* were assessed in the study. They were identified by morphology under microscope, Gram staining and using specific media for their culture. Results expressed as CFU/mL; percentage reduction calculated as: [(baseline CFU - 72-hour CFU) / baseline CFU] x 100. Biochemical Analysis: Serum samples (baseline and 2 months) and crevicular fluid (deepest pocket site, baseline and 2 months) were collected and analyzed via ELISA for IL-1β, TNF-α, CRP (Quantikine ELISA; R & D Systems) and ALP. Results expressed in pg/mL (IL-1β, TNF-α), mg/L (CRP) and U/L (ALP). Wound Healing and Safety Assessment: wound healing was assessed as reduction in clinical signs of gingival inflammation for all the three groups. The healing was considered adequate if the gingival health is returned with signs of stippling as well as reduction in bleeding on probing. The patients were also asked a safety questions and documented for staining, taste alteration, burning, irritation and allergic reactions at each visit. Oral soft-tissue examination was performed at baseline, 1 week and 2 months.

## Statistical Analysis:

Kruskal-Wallis test was performed with post-hoc Dunn-Bonferroni test to assess for between-group differences. Wilcoxon signed-rank test evaluated within-group changes. One-way ANOVA with Bonferroni correction assessed parametric biomarkers. Significance level was set at p=0.05 (two-tailed).

## Results:

All groups demonstrated significant reduction (HA: 1.68 ± 0.38 → 0.84 ± 0.21, 50% reduction; CHX: 1.74 ± 0.31 → 0.84 ± 0.21, 52%; SP: 1.70 ± 0.35 → 0.86 ± 0.19, 49%; control: 1.66 ± 0.42 → 0.92 ± 0.28, 44%; all p = 0.001) in Plaque index. No significant between-group differences (p = 0.523). Significant reduction in all groups was observed (HA: 34% reduction; CHX: 42%; SP: 40%; control: 36%; all p = 0.001). CHX and SP both demonstrated significantly greater GI reduction versus HA (p = 0.026 and p = 0.041, respectively), with no significant difference between CHX vs. SP (p = 0.681) was seen in Gingival index. Comparable reduction across groups (HA: 0.96 ± 0.08 mm; CHX: 1.04 ± 0.08 mm; SP: 1.00 ± 0.09 mm; control: 0.90 ± 0.12 mm; p = 0.147) was seen in Probing pocket depth. Similar CAL gain across groups (HA: 1.00 ± 0.33 mm; CHX: 1.10 ± 0.21 mm; SP: 1.04 ± 0.28 mm; control: 1.00 ± 0.31 mm; p = 0.681). *P. Gingivalis* reduction at 72 hours: HA 75% > CHX 37.5% > SP 42% > control 20% (p < 0.05). HA demonstrated significantly superior antimicrobial efficacy versus all comparators. Similar patterns observed for *F. nucleatum* (HA 75%, CHX 38%, SP 40%, control 14%) and *T. forsythia* (HA 75%, CHX 33%, SP 38%, control 11%). Total anaerobic count reduction: HA 68% > CHX 45% > SP 48% > control 18% was seen in Microbiological outcomes. The study assessed pro-inflammatory biomarkers and wound healing outcomes, as well as adverse effects associated with different treatments. Serum IL-1β showed a 20.3% reduction for HA, 13.0% for CHX, 28.6% for SP, and 20.8% for the control. SP demonstrated a significantly greater reduction in IL-1β compared to HA (p = 0.041), with no significant difference between CHX and SP (p = 0.147). For serum TNF-α, CHX had a 24.5% reduction, SP 22.4%, HA 8.1%, and the control 6.3%. Both CHX and SP were superior to HA and the control, though the differences between CHX vs. HA (p = 0.074) and SP vs. HA (p = 0.089) were not statistically significant. In serum CRP levels, HA showed a 35.0% reduction, CHX 40.7%, SP 38.4%, and the control 30.1%, with no significant intergroup differences (p = 0.283). Serum ALP showed a 13.4% reduction for CHX, 12.2% for SP, 7.0% for HA, and a -1.0% reduction for the control. Both CHX and SP were superior to the control (p < 0.05). In terms of wound healing, early epithelialization at 1 week was 92% for HA, 89% for SP, 68% for CHX, and 56% for the control, with HA and SP being significantly superior to CHX and the control (p = 0.015). Regarding adverse effects, HA had 4 patients (16%) experience transient mild burning, CHX had 8 patients (33%) report extrinsic staining, 6 (25%) taste alteration, and 3 (12%) mild burning, SP had 2 patients (8%) with transient mild burning and 1 (4%) with gingival sensitivity, while the control had 1 patient (4%) with sensitivity. SP demonstrated significantly fewer adverse effects than CHX (p = 0.018). No serious adverse events occurred in any group (Table 1).

## Discussion:

All adjunctive therapies and SRP alone achieved significant clinical improvement with comparable PPD and CAL reduction, consistent with meta-analytical evidence that mechanical instrumentation dominates early-stage of periodontal healing [6]. However, GI reduction demonstrated mechanistic differentiation: CHX and SP both achieved superior gingival inflammation improvement versus HA, suggesting that direct antimicrobial suppression of inflammatory pathogens and anti-inflammatory mechanisms outperform regenerative approaches alone in acute plaque-induced gingivitis [7]. HA has shown highest reduction of *P. Gingivalis* which was statistically significant as compared to the other groups. This emphasizes the role of HA in bacterial reduction which has had conflicting literature in the past. HA had been shown to be an effective adjunct to SRP [8]. The healing capacity of HA was found to be superior as compared to the other test groups, suggesting optimal soft tissue response. This could also serve as an adjunct to post-surgical healing [9]. CHX had shown statistically significant reduction of GI (42%) than HA (34%) and similar trend of TNF-α suppression (24.5% vs. HA 8.1%). The meta-analytic literature has confirmed these findings where CHX has outperformed SRP alone group in terms of PPD reduction [10]. These confirm the potency of CHX as an antimicrobial agent however; the adverse effects (33% staining, 25% taste alteration, 12% burning) were also substantially higher than those found for HA group. A recent study has shown that this could affect patients' preference [11]. *Salvadora persica* has alkaloids in its phytochemical composition which is responsible for its antimicrobial properties. It also has tannins and flavonoids which imparts it as antioxidant and anti-inflammatory properties [12]. This was observed in the result of the present study where SP has shown comparable results showing potent *P. Gingivalis* reduction (42%) and IL-1β reduction (28.6%). The adverse effects were lesser than the other two test groups too suggesting better acceptance in the patients. This was in accordance with the previous studies which favor SP's effectiveness in reducing plaque and gingivitis and promoting gingival healing [13]. This trial design was balanced; the therapeutic effects exerted by different treatment modalities which can be considered multi mechanism rather than single mechanism products validates the study's four-arm design and eliminates prior methodological bias inherent in HA vs. CHX comparisons. The limitations include short follow up period and smaller sample size.

## Conclusion:

Within the limitation of this prospective trial, there have been observed as comparable benefits of the HA, CHX and SP gels in Stage I-II chronic periodontitis patients. The herbal agent *Salvadora persica* has shown antimicrobial and anti-inflammatory efficacy with superior tolerability. Hyaluronic acid has shown better wound healing properties and antimicrobial potency. Chlorhexidine provides rapid anti-inflammatory benefits but with substantial adverse effects limiting routine applicability. Based on the result of this study, clinician can make a case-selective choice.

## Figures and Tables

**Table 1:** Comparative treatment outcomes summary

**Outcome**	**HA group**	**CHX group**	**SP group**	**Control group**	**pvalue / note**
GI reduction (%)	34	42	40	36	0.026 (CHX, SP > HA)*
BOP reduction (%)	79	82	82	72	0.087 (NS)
PPD reduction (mm)	0.96	1.04	1	0.9	0.147 (NS)
*P. Gingivalis* reduction (%)	75	37.5	42	20	<0.05 (HA > all)
IL-1β reduction (%)	20.3	13	28.6	20.8	0.041 (SP > HA)*
TNF-α reduction (%)	8.1	24.5	22.4	6.3	0.074 (trend CHX/SP > HA)
Early epithelialization at 1 week (%)	92	68	89	56	0.015 (HA, SP > CHX, Control)*
Patients with any adverse effect (%)	16	70	12	4	0.018 (CHX >> HA/SP/Control)*
*Statistically significant;
†Superior to comparators
